# Association between visceral adiposity and generalized anxiety disorder (GAD)

**DOI:** 10.1186/s40359-024-01542-x

**Published:** 2024-01-25

**Authors:** Ghazaleh Nameni, Shima Jazayeri, Masoud Salehi, Ali Esrafili, Ahmad Hajebi, Seyed Abbas Motevalian

**Affiliations:** 1 Department of Nutrition, School of Public Health, Iran University of Medical Sciences , Tehran, Iran; 2 Department of Biostatistics, School of Public Health, Iran University of Medical Sciences, Tehran, Iran; 3 Department of Environmental Health Engineering, School of Public Health, Iran University of Medical Sciences, Tehran, Iran; 4 Research Center for Addiction & Risky Behaviors (ReCARB), Psychosocial Health Research Institute, Iran University of Medical Sciences, Tehran, Iran; 5 Department of Epidemiology, School of Public Health, Iran University of Medical Sciences, Tehran, Iran

**Keywords:** Generalized anxiety disorder, GAD, Visceral adiposity, Cross-sectional

## Abstract

**Background and objectives:**

Due to an increased rate of inflammation in generalized anxiety disorder (GAD), insight into the mediating factors in the onset and recurrence of the inflammatory response can help to achieve novel treatments for alleviating the risk of GAD. In the current study, we aimed to evaluate the possible relationship between visceral adipose tissue (VAT) as an important intermediary in inflammation pathways and GAD in participants of the Employees’ Health Cohort Study of Iran (EHCSIR).

**Method:**

We analyzed the data from 3889 included participants aged > 18 years in the EHCSIR study, which were collected from 2017 to 2020. Lifetime and 12-month GAD were assessed using the Composite International Diagnostic Interview (CIDI-2.1) questionnaire. The adjusted prevalence ratio was computed to evaluate the association between GAD and visceral adiposity index (VAI), GAD and visceral fat area (VFA), GAD and body mass index (BMI) and ultimately GAD and waist circumference (WC) in males and females using STATA software.

**Results:**

Log-binomial analysis showed a higher prevalence ratio of 12-month GAD associated with VFA in women [PR: 1.42, CI: 1.07–1.87, P: 0.015]. The prevalence of lifetime GAD was higher in obese women (BM1 > 30) [PR: 2.35, CI: 1.07–5.13, P:0.03] than in women with normal BMI. Women with higher VAI were also significantly more likely to suffer lifetime GAD [PR: 1.25, CI: 1.05]. 1.48, P:0.01]. In males, the prevalence of lifetime diagnosed GAD per 1 standard deviation increase in VFA was 0.65 [CI: 0.46–0.91, P: 0.01].

**Conclusion:**

Visceral adiposity as a positive agent was associated with GAD prevalence in women. The presence of GAD symptoms showed no relationship to VFA in men.

## Introduction

“Sixth leading cause of global disability belongs to anxiety disorders” is a result of the Global Burden of Disease study [1]. As a pervasive problem, anxiety disorders are common among mental disorders in the general population in the past two decades [2]. The acceleration of the obesity phenomenon as one of the major public health challenges has also been mentioned repeatedly globally. However, not every obese person develops chronic complications, and this heterogeneity is the missing link in the decisions of physicians. A bidirectional relationship between anxiety disorders and obesity has been reported more recently, especially in adolescence, as a principal period for physical and psychological development [3]. While some studies support the association between depressive mood and visceral adipose tissue (VAT) and waist circumference (WC) [4–7] and body mass index (BMI) as a proxy for adiposity with mood [8], other studies have mentioned that higher BMI is associated with fewer mood disorders [9–13]. Even, I. Hach et al point to the invalidity of global supportive studies in relation to the interrelationship of obesity and mental health problems and recommended further studies to evaluate the possible link between specific aspects of obesity and the other psychopathology subtypes [14].

The most common subtype of anxiety disorders, generalized anxiety disorder (GAD), is defined by continuous patterns of worrying, tension, anxiety, mindfulness and emotion regulation difficulties. Memory and cognitive dysfunction also usually occur in constant GAD symptoms that lead to dysregulations in social, personal and educational performance. The highest prevalence of GAD is in middle-aged people, and patients suffering GAD symptoms have higher health costs by 64% compared with those without GAD, and comorbid GAD conditions have complicating and costly effects on over half of all mental and medical situations [2]. Furthermore, investigations of various subtypes of anxiety disorders in two studies showed no significant relationship between BMI and GAD, unlike specific phobia, panic disorders and social phobia [15, 16]. In contrast, other studies showed a positive significant association between these two conditions [8, 17]. In other words, previous investigations have almost consistently reported an association between an increased rate of inflammation in anxiety [18–20] and GAD [21]. Accordingly, insight into the mediating factors in the onset and recurrence of the inflammatory response can help to achieve novel treatments for alleviating the risk of GAD. Among adipose depot sites, visceral adipose tissue (VAT) with a pathogenic fat depot [21] secretes higher concentrations of inflammatory cytokines. Cannavale, C. N et al. demonstrated that central obesity via systemic inflammatory markers was linked to negative effects on behavioral and neuroelectric markers of attentional inhibitory control [22]. Indeed, it has been reported that structural and functional defects of the brain are induced by VAT [23]. Accordingly, the role of VAT may be recognized as an important factor in the development of GAD.

Taken together, due to the considerable health burden of GAD and the striking inconsistency in findings of the interrelationship between obesity and GAD, the current study provides a basis of investigation to identify the “visceral adiposity signature” in association with GAD patients.

## Method

### Participants

In the current study, we used data from the Employees Health Cohort Study of Iran (EHCSIR), a cohort study designed to assess the risk factors for non-communicable diseases among Iranian public sector employees affiliated with the Ministry of Health and Medical Education. Among 4886 participants aged 18 years and older who were recruited from July 2017 to February 2020, 3889 participants with complete data were included in the present study. Written informed consent was obtained from all participants, and the Ethical Committee of IUMS approved the study (IR.IUMS.REC.1400.048).

### Generalized anxiety disorder assessment

The validated Persian version of Clinical interview CIDI (Composite International Diagnostic Interview) (2.1 CIDI based on DSM-IV) [24, 25] as a gold standard questionnaire for the diagnosis of mental disease according to the Diagnostic and Statistical Manual of Mental Disorders, 4th edition (DSM-IV) and International Classification of Diseases, 10th revision (ICD-10) criteria was used for all participants. The CIDI is an organized practical diagnostic interview with good reliability and validity that has been widely used in studies [26, 27]. Diagnosis of generalized anxiety disorder (GAD) by this questionnaire has been generated in life-time and 12-month periods. Trained psychologists managed CIDI interviews and considered persons in the GAD group if they reported symptoms related to excessive anxiety, which is difficult to regulate, along with clinical signs according to CIDI assessment criteria.

### Measures of visceral adiposity

For this, we used two methods: visceral adiposity index (VAI) and BIA (Bioelectrical Impedance Analysis) tools because of their simplicity, applicability and noninvasiveness.

VAI as a reliable replacement of MRI was calculated as (WC/(36.58 + (1.89 × BMI)) × ((TG/0.81) × (1.52/HDLC)) for women and (WC/(39.68 + (1.88 × BMI)) × ((TG/1.03) × (1.31/HDLC)) for men [28]. Anthropometric measures covering weight (using InBody 770), height (using wall-mounted measuring tape) and waist circumference (WC) (using measuring tape at the midpoint between the lowest rib and the anterior superior iliac crest) were accomplished in the fasting situation with an empty bladder. Specific cutoff points of WC were considered as 99.5 cm for men and 94.25 cm for women according to the desired criteria for the Iranian population [29]. HDL and TG were measured by a Biotecnica BT 1500, and BMI was measured by dividing the weight by the square of the height (kg/m^2^).

InBody 770 was used to specify body composition. The results of BIA-based methods are comparable to CT scans as a reference. Participants feet touched floor electrodes while standing and were asked to snatch the electrodes by their hands. When their legs were apart and two arms were raised 45° away from the body, InBody checked the impedance of the trunk and limbs. Finally, the results of the visceral fat area (VFA) were used. Details have been reported previously [30].

### Covariates

After clinical and generalized interviews, all participants had complete data about demographic properties, sex, age, marital status, education, job, socioeconomic status, smoking and medical history, including physical and mental illness. Physical activity was assessed by the short version of the International Physical Activity Questionnaire (IPAQ), which reports physical activity results according to continuous values of MET minutes in a week (MET = metabolic equivalent of task).

### Statistical analysis

Demographic and metabolic variables in participants with 12-month and lifetime GAD symptoms were described by counts as percentages. The strength of the association between generalized anxiety disorder groups (12-month and lifetime GAD) and VAI, VFA, BMI, WC analyzed by LOGLINK (log-binomial analysis) and reported in adjusted prevalence ratio with its CI of 95% ratio. Confounding factors: age, marital status, smoking, educational level, socioeconomic status, physical activity, and prevalence of chronic diseases, including diabetes, hypertension and cardiovascular disease, were adjusted in two models. For all statistical analyses, we used STATA (version 12.1) statistical software.

## Results

### Demographic characteristics

Participants (mean age 42.7 yrs.) were divided into generalized anxiety disorder (GAD) in 12 months and a lifetime group (i.e., the control group) according to the CIDI questionnaire; 109 of 3889 included participants were classified into 12-month GAD with 38 males and 71 females, and 224 were in a lifetime GAD group confined to 63 male and 161 female participants. Other demographic characteristics of the participants are presented in Table 1.

**Table 1:** Baseline prevalence of 12-month and life-time generalized anxiety disorder (GAD) by sociodemographic characteristics

Characteristics	Total		12-month Generalized Anxiety Disorder		P-value	Life-time Generalized Anxiety Disorder	P-value
			(*N* = 109)			(*N* = 224)	
**Gender: N (%)**							
Male	1439		38 (2.64)		0.65	63 (4.38)	0.005
Female	2460		71 (2.89)			161 (6.54)	
**Age group (years): N (%)**							
34 or less	622		17 (2.73)		0.7	32 (5.14)	0.4
35–44	1708		47( 2.75)			91 (5.33)	
45–54	1254		39 (3.11)			80 (6.38)	
55 or more	315		6 (1.9)			21 (6.67)	
**Educational level: N (%)**							
Primary or Secondary	356		7 (1.97)		0.47	5.9	0.27
High school diploma	767		25 (3.26)			6.91	
Associate degree	365		14 (3.84)			7.4	
Bachelor’s degree	1470		43 (2.93)			5.37	
Master’s degree	737		16 (2.17)			4.61	
PhD/specialty	196		4 (2.04)			4.59	
**Job: N (%)**							
Office work	1248		47 (3.77)		0.06	86 (6.89)	0.05
Academic	111		1 (0.9)			5 (4.5)	
Health professional	1809		43 (2.38)			85 (4.7)	
Service work	713		18 (2.52)			47 (6.59)	
**Marital status: N (%)**							
Married	3089		82 (2.65)		0.59	167 (5.41)	0.01
Never married	524		16 (3.05)			30 (5.73)	
Ex-married	214		8 (3.74)			22 (10.28)	
**Wealth index: N (%)**							
Low	977		22 (2.25)		0.19	61 (6.24)	0.40
Middle	1958		64 (3.27)			114 (5.82)	
High	922		22 (2.39)			46 (4.99)	
**Coronary artery disease: N (%)**							
Yes	78		5 (6.41)		0.04	8 (10.26)	0.07
No	3555		93 (2.62)			199 (5.60)	
**Hypertension: N (%)**							
Yes	320		92 (2.78)		0.53	21 (6.56)	0.50
No	3307		7 (2.19)			186 (5.62)	
**Diabetes mellitus: N (%)**							
Yes	188		6 (3.19)		0.69	12 (6.38)	0.60
No	3447		93 (2.70)			196 (5.69)	
**Tobacco smoking: N (%)**							
Yes	458		15 (3.28)		0.42	32 (6.99)	0.20
No	3201		84 (2.62)			177 (5.53)	
**Adequate physical activity: N (%)**							
Yes	1304		36 (2.76)		0.85	87 (6.67)	0.11
No	2157		62 (2.87)			116 (5.38)	

### Association between GAD and anthropometric variables

The association between 12-month and lifetime GAD groups with visceral adiposity, BMI and WC using log-binomial analysis was performed after adjusting for age, sex, marital status, smoking status, educational level, job, socioeconomic status, physical activity, and prevalence of chronic diseases, including diabetes, hypertension and cardiovascular disease **(**Tables 2 and 3**)**. Within each sex, the prevalence ratio of GAD in women per 1 standard deviation increase in VFA was 1.42 [CI: 1.07–1.87, P: 0.015] in the 12-month GAD fully adjusted model. Similarly, the increase in the VAI was associated with an increased likelihood of 12-month GAD in women. However, this association was not statistically significant. Indeed, the prevalence of 12-month GAD was higher in obese women (BM1 > 30) [PR: 2.35, CI: 1.07–5.13, P:0.03] than in women who were overweight compared with women with normal BMI. Women with higher VAI were also significantly more likely to suffer lifetime GAD [PR: 1.25, CI: 1.05]. 1.48, P:0.01]. In males, the prevalence of lifetime diagnosed GAD per 1 standard deviation increase in VFA was 0.65 [CI: 0.46–0.91, P: 0.01], and a higher visceral fat area in men showed a protective effect against GAD prevalence.

**Table 2:** Adjusted prevalence ratios (APRs) of 12-months feneralized anxiety disorder (GAD) regarding to adiposity measurements

Variables	Men				Women			
	model 1		model 2		model 1		model 2	
	APR(95%CI)	P	APR(95%CI)	P	APR(95%CI)	P	APR(95%CI)	P
**VAI** (per 1 SD)	0.83 (0.55, 1.25)	0.38	0.81 (0.51, 1.28)	0.38	1.05 (0.94, 1.16)	0.37	1.25 (0.96, 1.62)	0.09
**VFA** (per 1 SD)	0.95 (0.66, 1.36)	0.79	0.76 (0.49, 1.18)	0.22	1.36 (1.06, 1.74)	0.01	1.42 (1.07, 1.87)	0.01
**Overweight** (vs. normal)	0.82 (0.38, 1.76)	0.61	0.65 (0.29, 1.46)	0.30	1.59 (0.87, 2.93)	0.12	1.85 (0.90, 3.78)	0.08
**Obese** (vs. normal)	0.78 (0.32, 1.93)	0.60	0.66 (0.25, 1.73)	0.40	1.86 (0.94, 3.67)	0.07	2.35 (1.07, 5.13)	0.03
**WC** (high vs. low)	1.05 (0.53, 2.09)	0.87	0.77 (0.34, 1.71)	0.52	1.52 (0.89, 2.58)	0.12	1.63 (0.89, 2.94)	0.1

**APR**: Adjusted Prevalence Ratio per 1 SD increment in adiposity measures associated to: VAI, VFA, BMI (obese and overweight vs. normal weight) and WC (high vs. low)

**Normal weight** (BMI 18.5–24.9), **overweight** (BMI > 25), **obese** (BMI > 30); weight was analyzed according to BMI category

**WC** men/women (99.5/94.25); WC was analyzed according to cut-off points in men and women

**VAI**: visceral adiposity index, **VFA**: visceral fat area (cm^2^), **BMI**: body mass index (kg/m^2^), **WC**: waist circumference (cm)

**CI**: Confidence Interval; **Model 1**: adjusted by age, socioeconomic status, marital status, education, job; **Model 2** (full adjusted: in addition to variables adjusted in model 1, smoking, physical activity and chronic disease like diabetes, hypertension and cardiovascular disease were added

**Table 3:** Adjusted prevalence ratios (APRs) of life-time generalized anxiety disorder )GAD) regarding to adiposity measurements

Variables		Men					Women			
		model 1		model 2			model 1		model 2	
		APR(95%CI)	P	PR(95%CI)		P	APR(95%CI)	P	APR(95%CI)	P
**VAI** (per SD)		0.93 (0.71, 1.21)	0.59	0.94 (0.71, 1.25)		0.69	1.03 (0.96, 1.10)	0.34	1.25 (1.05, 1.48)	0.01
**VFA** (per SD)		0.81 (0.61, 1.08)	0.16	0.65 (0.46, 0.91)		0.01	1.05 ( 0.89, 1.24)	0.54	1.035 (0.86, 1.23)	0.69
**Underweight** (vs. normal)		-		-			1.90 (0.48, 7.47)	0.35	2.11 (0.53, 8.29)	0.28
**Overweight** (vs. normal)		0.85 (0.48, 1.52)	0.59	0.82 (0.44, 1.55)		0.56	1.25 (0.85, 1.83)	0.25	1.25 (0.82, 1.90)	0.29
**Obese** (vs. normal)		0.61 (0.29, 1.26)	0.18	0.48 (0.21, 1.13)		0.09	1.34 (0.87, 2.07)	0.17	1.35 (0.85, 2.16)	0.19
**WC** (high vs.low)		0.85 (0.49, 1.48)	0.58	0.64 (0.33, 1.26)		0.20	1.23 (0.87, 1.75)	0.23	1.22 (0.83, 1.79)	0.29

**APR**: Adjusted Prevalence Ratio per 1 SD increment in adiposity measures associated to: VAI, VFA, BMI (obese and overweight vs. normal weight) and WC (high vs. low)

**Normal weight** (BMI 18.5–24.9), **underweight** (BMI < 18.5), **overweight** (BMI > 25), **obese** (BMI > 30); weight was analyzed according to BMI category

**WC** men/women (99.5/94.25); WC was analyzed according to cut-off points in men and women

**VAI**: visceral adiposity index, **VFA**: visceral fat area (cm^2^), **BMI**: body mass index, **WC**: waist circumference (cm)

**CI**: Confidence Interval; **Model 1**: adjusted by age, socioeconomic status, marital status, education, job; **Model 2** (full adjusted: in addition to variables adjusted in model 1, smoking, physical activity and chronic disease like diabetes, hypertension and cardiovascular disease were added

## Discussion

Among communities in the cohort study of EHCSIR in which we investigated the relationship between generalized anxiety disorder (GAD) and visceral adiposity using the visceral adiposity index (VAI) and bioelectrical impedance analysis (BIA) compared to each other, a significant association between the 12-month GAD group and visceral fat area (VFA) measured by InBody in women was observed. Moreover, the prevalence of lifetime GAD was significantly associated with VAI only in women. Elevated 12-month GAD symptoms were also associated with obesity in women. Interestingly, this significant association was not observed in the relationship between visceral adiposity and GAD in men, and we even observed a protective effect of VFA against GAD in male participants.

Visceral adipose tissue (VAT), as a fat depot with adverse metabolic consequences, is considered an important intermediary in inflammation and oxidative stress pathways. In contrast, subcutaneous adipose tissue (SAT) has been stated as a potential protective fat depot in two animal studies [31, 32]. Some studies also reported that the adverse effect of SAT correlated with the amount of VAT [33]. Indeed, BMI cannot be considered a body fat distribution indicator, and interestingly, significant differences between BMI and health consequences in various familial groups have been reported. Therefore, we used BIA as a clinical practice [34] index to assess the amount of VAT.

The available studies that provide anthropometric criteria related to adiposity have focused on other mood disorders, including depression. A cross-sectional population-based study of 1284 included participants aged 50–70 years, and a possible relationship between BMI and visceral obesity (measured by waist circumference (WC) and waist-to-hip ratio (WHR)) with depressive symptoms was investigated. WHR and specifically WC were significantly associated with somatic-affective symptoms. In addition, BMI was significantly associated with somatic and cognitive-affective symptoms [4]. In older populations aged between 70 and 79 years, VAT was mentioned as a trigger of depressive mood only in men, in contrast to the present results [35]. In the literature in 1,581 women and 1,718 men, no association was observed between VAT and SAT and Center for Epidemiologic Studies Depression (CES-D) score in men. Conversely, according to adjusted BMI and age, the odds ratio of depressive symptoms per standard deviation increase in VAT was 1.33 times higher in women [6].. In a study of pre- and postmenopausal overweight and obese women, a strong association between depression symptoms and VAT was reported [36].

Despite several investigations that have assessed the link between obesity and anxiety disorders, inconsistent results have been reached. Specifically, research regarding GAD and adiposity is weak and even numerable. Contrary to our results, Gomes, Ana Paula et al. in their cohort study reported no association between the incidence of GAD and any obesity measure (measured by fat mass index (FMI), body mass index (BMI) and WC) in 2977 participants who were followed up for 18–22 years [37]. Other evidence with similar results based on no bidirectional association between GAD and BMI was presented by Pickering, RP et al. [38]. According to Tantawy, S et al., a significant negative correlation between BMI and GAD was reported in male and female students aged between 18 and 25 years. The limitations of this study, small sample size, not considering obesity criteria in addition to BMI and deficiency of participants in the underweight or obesity range, are mentioned [39]. A meta-analysis on 13 cross-sectional findings in which only two studies assessed GAD patients mentioned a positive significant association between anxiety disorders and BMI, although those relationships may be related to other types of anxiety disorders, such as specific phobia, panic disorder, social phobia, obsessive compulsive disorder, posttraumatic stress disorder or agora-phobia [40]. Another study using Mendelian randomization (MR) reported that an increased amount of adipose tissue leads to GAD. Nonlinear MR provided evidence of a significant relationship between a unit higher BMI and 1.37 higher odds of current GAD only in women [41].

Several hypotheses for the commentary of association between anxiety/depressive moods and percentage of body fat exist. The regulation of various substances, such as neuroendocrine and behavioral factors, including physical activity, energy intake, insulin and leptin, and especially the hypothalamic-pituitary adrenal (HPA) axis, is controlled by the body in different conditions. The potential overlap exists in the overactivity of the HPA axis, the increase in cortisol secretion and the occurrence of anxiety. In turn, elevated cortisol is associated with visceral obesity and loss of muscle mass, and overactivity of the HPA favors accumulation of visceral adipose tissue [42]. In the present investigation, we found an interaction related to the relationship between visceral adiposity and GAD in the two sexes, and we showed this association in women but not in men. Among biological mechanisms, HPA axis dysregulation plays a more important role in women than in men because of more abdominal obesity in women, which leads to alterations in the central and peripheral functions of the HPA axis [3]. The other reason for the sex differences in the pathogenesis of anxiety disorders is attributed to testosterone, which is present at approximately 10 times lower concentrations in women. The effects of testosterone on different regions of the brain are mediated by stimulation of estrogen receptors, androgen receptors, or GABA receptors. Additionally, testosterone suppresses activation of the HPA axis. In general, the alleviating effect of testosterone on mood symptoms and the protective effect of testosterone on mood disturbance in both genders have been reported several times [43, 44]. Indeed, simultaneously with the increase in VAT and consequent hypothalamic inflammation, testosterone release is impaired. Catherine Kim et al. suggested that the effect of VAT on testosterone concentration was more significant than that of SAT [45]. As such, the difference in the attitude of women in relation to their ideal figure exposes them to more emotional distress compared to men. Therefore, 30% of males prefer overweight figures, and generally, females experience more concern about their body shape and body weight [46].

On the basis of prior reported findings that lower HDL and higher TG levels were associated with improved mood and tension anxiety [47] and according to our findings related to the nonsignificant association between the prevalence of 12-month GAD and VAI, we suggest that VAI application in association with GAD and other related disorders should be performed with caution. Reasonable use of the VAI in this area can be beneficial in most studies.

In summary, GAD was associated with higher visceral adiposity among adolescent women. The fact that we discovered a significant positive association between lifetime GAD and visceral fat area (InBody) but not between 12-month GAD and VAI reflects the differences between the results of these two methods in measuring adipose tissue or that there is an intervening agent that affects the final consequence. Despite emphasizing that our findings emphasize the significance of the distribution of adipose tissue in relation to GAD, future studies are needed to support our results. Additionally, we suggest Mendelian randomization analysis and the use of gene variants to assess the causal effect of the present findings in further studies.

### Strengths and limitations

As a limitation, this study could not clarify the cause‒effect relationship because of its cross-sectional design; therefore, the direction of the present association should be investigated in longitudinal studies. The most significant strength of our study is the use of valid diagnostic tools to measure GAD. Indeed, our study consists of a large sample size with both men and women in different BMI categories and uses two methods for measuring visceral adiposity and objective measures for essential cofounders that make an important portion of the investigations.

## Conclusion

Visceral adiposity was significantly positively associated with the presence of GAD in women. Our findings support the need to reduce visceral adiposity in women to diminish the related adverse consequences of GAD, and obese women need to be alert about the increased risk of this disorder.

## Data Availability

No datasets were generated or analysed during the current study.
